# A Bibliometric Analysis of Triptolide and the Recent Advances in Treating Non–Small Cell Lung Cancer

**DOI:** 10.3389/fphar.2022.878726

**Published:** 2022-05-30

**Authors:** Quancheng Yang, Xuejia Zhai, Yi Lv

**Affiliations:** Department of Pharmacy, Union Hospital, Tongji Medical College, Huazhong University of Science and Technology, Wuhan, China

**Keywords:** triptolide, CiteSpace, non–small cell lung cancer, signaling pathways, antitumor activity

## Abstract

In recent decades, natural products derived from plants and their derivatives have attracted great interest in the field of disease treatment. Triptolide is a tricyclic diterpene extracted from *Tripterygium wilfordii*, a traditional Chinese medicine, which has shown excellent therapeutic potential in the fields of immune inflammation and cancer treatment. In this study, 1,106 Web-of-Science-indexed manuscripts and 1,160 Chinese-National-Knowledge-Infrastructure-indexed manuscripts regarding triptolide published between 2011 and 2021 were analyzed, mapping the co-occurrence networks of keywords and clusters using CiteSpace software. The research frontier and development trend were determined by keyword frequency and cluster analysis, which can be used to predict the future research development of triptolide. Non–small cell lung cancer (NSCLC) is most common in lung cancer patients, accounting for about 80% of all lung cancer patients. New evidence suggests that triptolide effectively inhibits the development and metastasis of NSCLC by the induction of apoptosis, reversion of EMT, and regulation of gene expression. Specifically, it acts on NF-κB, MAPKs, P53, Wnt/β-catenin, and microRNAs (miRNAs), signaling pathways and molecular mechanisms. Consequently, this article reviews the research progress of the anti-NSCLC effect of triptolide. In addition, attenuated studies on triptolide and the potential of tumor immunotherapy are also discussed.

## 1 Introduction

**FIGURE 1:**
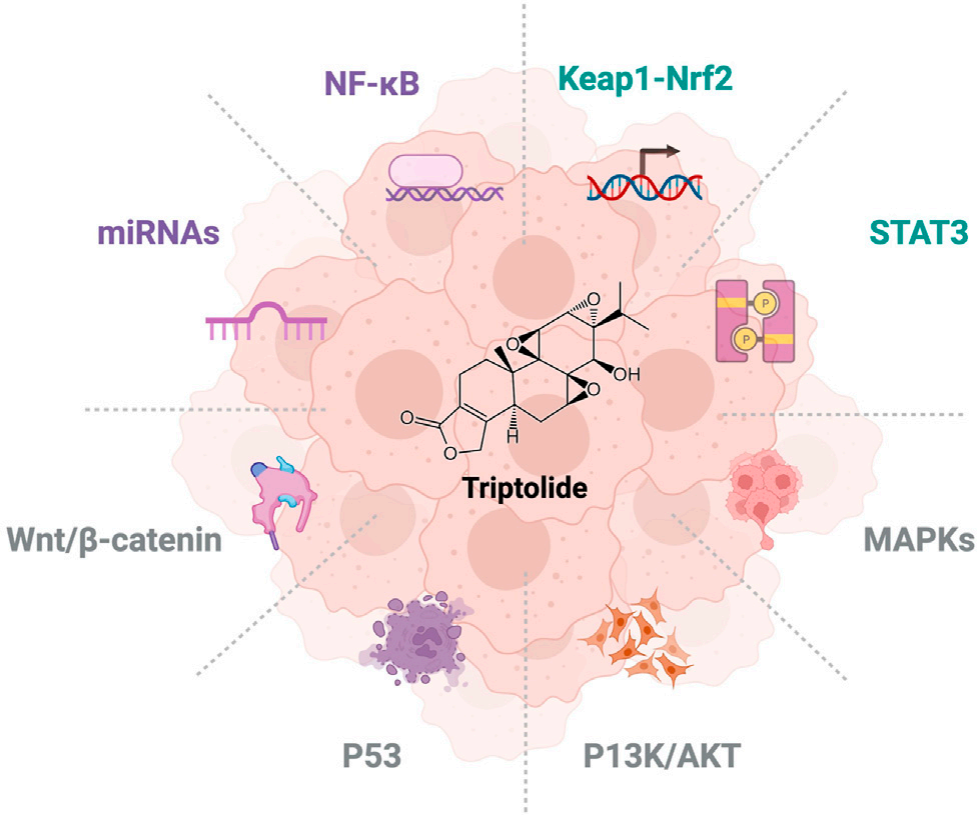
Overview of triptolide on signaling pathways for NSCLC research.

Triptolide is a diterpenoid tricyclic oxide (Noel et al., 2019), which was reported to be extracted and isolated from *Tripterygium wilfordii* Hook F in 1972 (Kupchan et al., 1972), as shown in Figure 1. Triptolide has a wide range of pharmacological activities including anti-rheumatism, antibacterial, anti-inflammatory, immunomodulatory and anti-tumor, and has significant inhibitory effects on breast cancer, prostate cancer, liver cancer, and other cancers. For decades, research based on the anticancer activity of natural products has attracted many researchers’ interest. Compared with classical chemotherapy drugs, natural products have stronger targeting, fewer side effects, and less resistance (Deng et al., 2020).

CiteSpace is a visualization software developed by Dr. Chen Chaomei for bibliometrics, which can be used to map scientific knowledge. Since its launch in 2004, this software has been widely used by relevant researchers in bibliometrics. The main program steps of CiteSpace are time slicing, threshold, modeling, pruning, merging, and mapping (Liu et al., 2019). It can also help users identify key nodes by identifying the centrality of nodes, and intuitively show research hotspots in a research field, key paths, and nodes of field evolution. This study intends to use CiteSpace to analyze the literature related to triptolide since 2011 and establish the co-occurrence network and clustering network of keywords.

The Global Burden of Cancer report shows that in 2017, trachea, bronchus, and lung cancer (TBL) were the most common cause of cancer deaths in men (approximately 1.3 million) and the leading cause of cancer deaths in women (approximately 600,000) (Global Burden of Disease Cancer et al., 2019). In recent years, many studies have shown that triptolide can inhibit proliferation, apoptosis, migration, and invasion of non–small cell lung cancer (NSCLC) (Wei et al., 2019). Triptolide exerts its anticancer effect through the NF-κB pathway (Lee et al., 2002; Jiang et al., 2016; Zheng et al., 2017), MAPK signaling pathway (Frese et al., 2003; Tai et al., 2010; Xie et al., 2016), regulation of microRNAs (Reno et al., 2015; Li et al., 2016; Philips et al., 2020), Wnt/β-catenin signaling pathways (Reno et al., 2016; Mao et al., 2018; Nardi et al., 2018), and other targets. Given the toxicity characteristics of triptolide *in vitro* and *in vivo*, some studies focused on the drug combination of triptolide. Low-dose triptolide can increase the sensitivity of NSCLC cells to chemotherapy with cisplatin and other drugs (Zhu et al., 2018) and overcome the resistance of NSCLC cells to gefitinib and other drugs (Tong et al., 2019; Li et al., 2020). This study systematically summarized the targets and molecular mechanisms of triptolide in NSCLC, and summarized the latest progress of triptolide combined therapy for NSCLC, as shown in Table 1.

**TABLE 1:** Mechanism of action of triptolide against non–small cell lung cancer.

Signaling pathways	Mechanism of action	Model/cell line	References
miRNAs	miR-21↓; caspase-3↑; caspase-9↑; PTEN protein↑	PC-9	Li et al. (2016)
	Cav-1↓; SIRT-1↓; miR204-5p↑; p-Akt↓	A549; H460	Philips et al. (2020)
	126miRNAs↑; 101miRNAs↓; FAK↓; MMP14↓; Src (Y416) ↓; p130Cas (Y247)↓	H460	Reno et al. (2015)
NF-κB	Reverse transcription activation of p65↓	A549; NCI-H1299	Lee et al. (2002)
	P65↓; FLIP↓; XIAP↓; Bcl-2↓; Bcl-xL↓; COX-2↓	A549/Taxol	Jiang et al. (2016)
	MMP-9↓; p-P65↓	A549; H460; NCI-H446	Mao et al. (2018)
	Nuclear NF-κB localization was blocked	H460	Zheng et al. (2017)
P53	P53↑	A549; H460; NCI-H446	Mao et al. (2018)
	Combination of RPL23 and MDM2↑; combination of P53 to MDM2↓; phosphorylation of P53↑	H460	Zheng et al. (2017)
	P53↑; activation of P38 α and ERK1/2↑; phosphorylation of P53↑	A549	Wang et al. (2020)
MAPKs	Activation of ERK2↑	A549; NCI-H358	Frese et al. (2003)
	p-p38↑; p-ERK↑; p-JNK↓	A549/Taxol	Xie et al. (2016)
	MKP-1↓	NCI-H441	Tai et al. (2010)
Wnt/β-catenin	E-cadherin↑; ZEB1↓; vimentin↓; slug↓; β-catenin↓	A549; NCI-H1299	Deng et al. (2021)
	β-catenin↓; Jagged1↓; c-Myc↓; p-p70S6K↓; p-GSK-3α↓; p-GSK-3β↓	A549/TaxR	Tian et al. (2021)
	WIF1 promoter methylation↓; WIF1↑; WIF1 mRNA↓; β-catenin↓; Axin2↓	A549; H460	Mao et al. (2018)
	Dimethylation and trimethylation of H3K4, H3K9, H3K27, H3K36, and H3K79↑; WIF1↑; FRZB↑; SFRP1↑; ENY2↑; DKK1↑	E160D FEN1 mouse model; NSG xenograft tumor mouse model	Nardi et al. (2018)
PI3K/Akt	p-GSK-3β↑; p-Akt↓	A549/Taxol	Xie et al. (2016)
CaMKKβ/AMPK	p-AMPK↑; p-Akt↓	A549; NCI-H1395	Ren et al. (2020)
STAT3	p-STAT3↓; C-myc↓; cyclin D1↓; MCL-1↓; BCL-2↓	PC-9; A549	Huang et al. (2019)
Keap1/Nrf2	NFE2L2 mRNA↓; GCLC mRNA↓; AKR1C1 mRNA↓; Nrf2↓	A549	Zhu et al. (2018)
CD44/RHAMM	HASs↓; HA↓; CD44↓; RHAMM↓	A549; NCI-H1299; H520	Song et al. (2017)

Cav-1, caveolin-1, SIRT-1, sirtuin-1, p-Akt, phosphorylated protein kinase B, FAK, focal adhesion kinase, MMP14, matrix metalloproteinase 14, FLIP, FLICE inhibitory protein, XIAP, X-linked inhibitor of apoptosis protein, RPL23, ribosomal protein L23, MDM2, murine double minute 2, MKP-1, mitogen-activated protein kinase phosphatase-1, ZEB1, zinc finger E-box-binding homeobox 1

## 2 Literature Analysis Based on CiteSpace

### 2.1 Data Source

The English literature was retrieved from the core collection database of Web of Science with the subject word “Triptolide.” The publication year was from January 1, 2011 to December 21, 2021, and 1,106 valid works of the literature were finally included after excluding irrelevant literature.

The Chinese literature was retrieved from the Chinese National Knowledge Infrastructure (CNKI) database with the subject word “Triptolide.” The publication year was from January 1, 2011, to December 21, 2021, and 1,160 valid works of the literature were finally included after excluding irrelevant literature.

### 2.2 Software and Parameters

CiteSpace 5.8. R3 was used for the analysis and time slice. From 2011 to 2021, each year is a time slice. Term source selection, node keyword threshold (top *N* = 50), pruning map with Pathfinder, pruning networks, pruning the merged network pruning algorithm, keyword co-occurrence analysis, and cluster analysis were carried out for triptolide.

### 2.3 Results

#### 2.3.1 Co-Authorship

**FIGURE 2:**
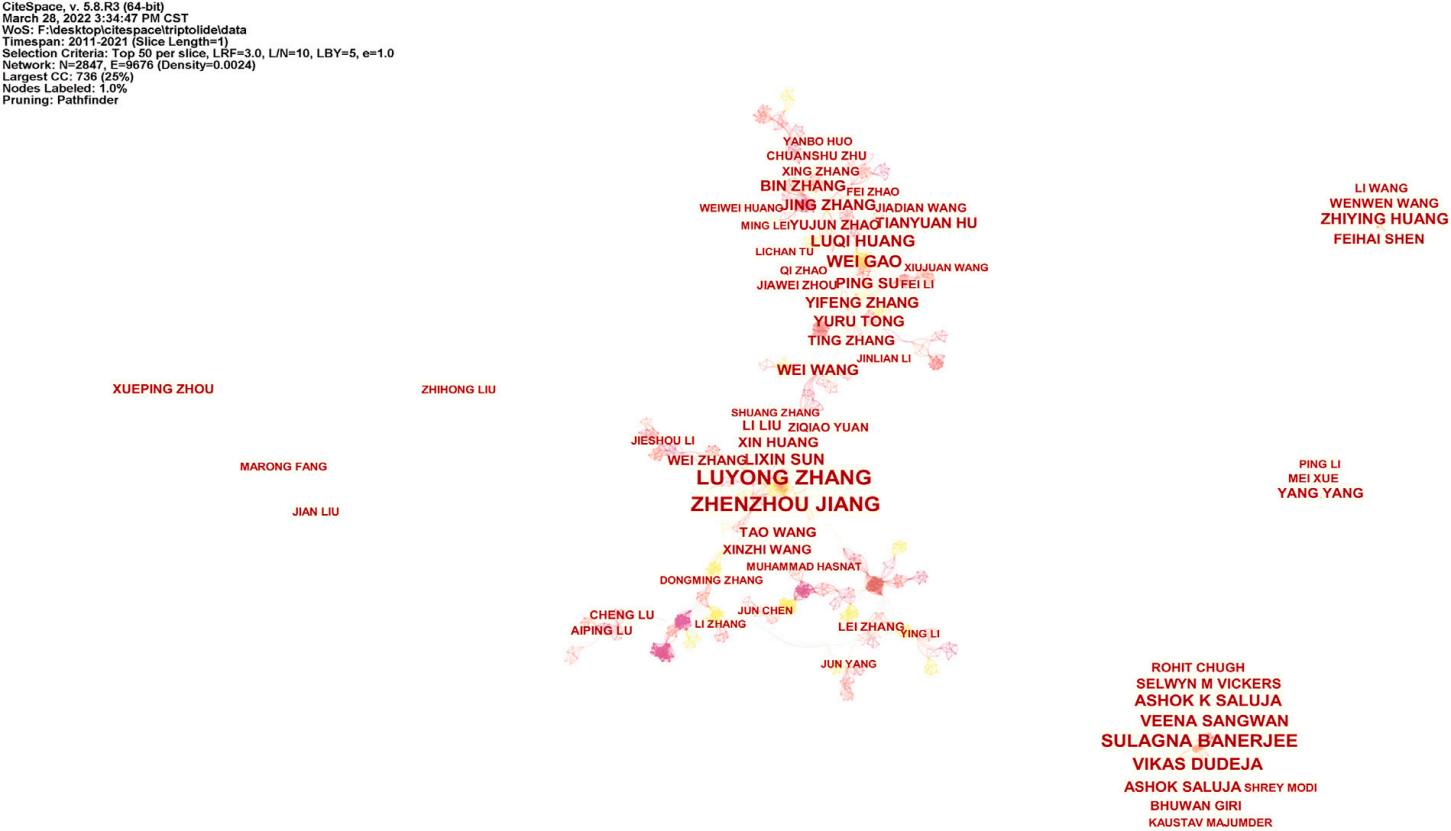
Co-authorship network of triptolide literature keywords from the WOS.

Studies published from 2011 to 2021 were analyzed over a one-year time slice. Figure 2, 3 show the co-authorship network results from WOS and CNKI literatures. The size of the circle represents the number of articles published by a country. The shorter the distance between the two circles, the greater the cooperation between the two countries will be.

**FIGURE 3:**
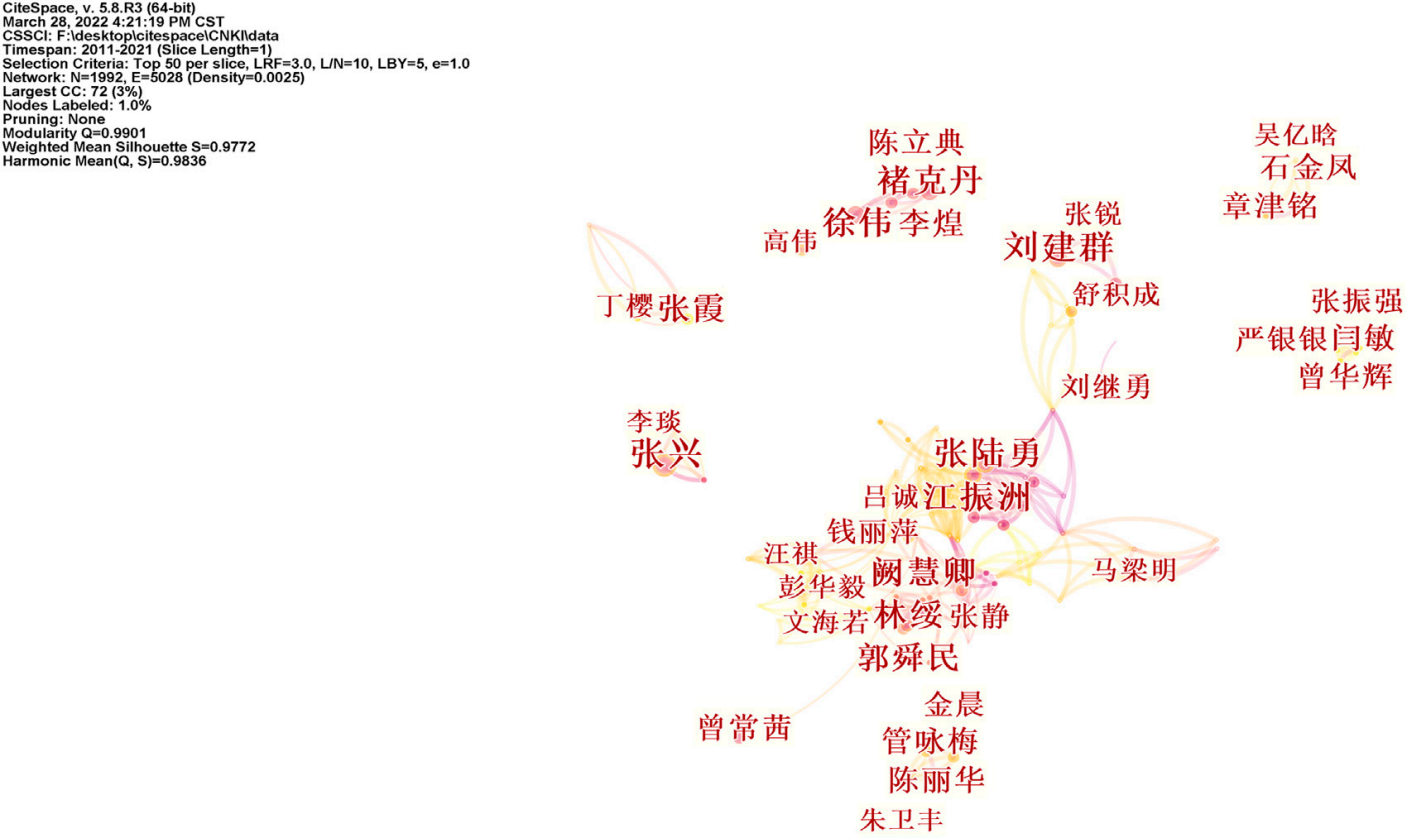
Co-authorship network of triptolide literature keywords from the CNKI.

#### 2.3.2 Co-Country

**FIGURE 4:**
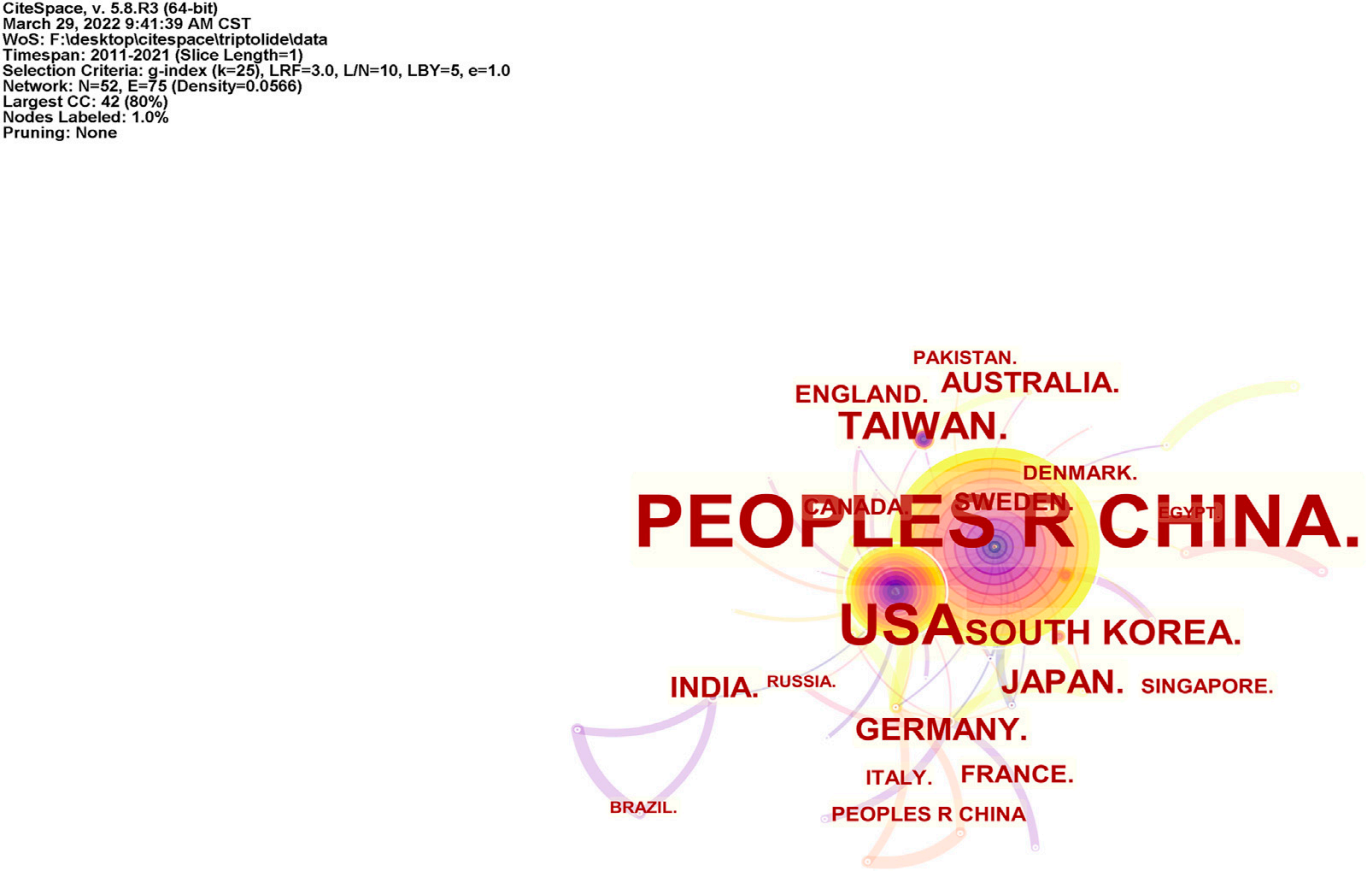
Co-countries network of triptolide literature keywords from the WOS.

Studies published from 2011 to 2021 were analyzed over a one-year time slice. Figure 4 shows the common country results in the WOS literature. The size of the circle represents the number of articles published by a country. The shorter the distance between the two circles, the greater the cooperation between the two countries will be.

#### 2.3.3 Co-Occurring Keyword Analysis

**FIGURE 5:**
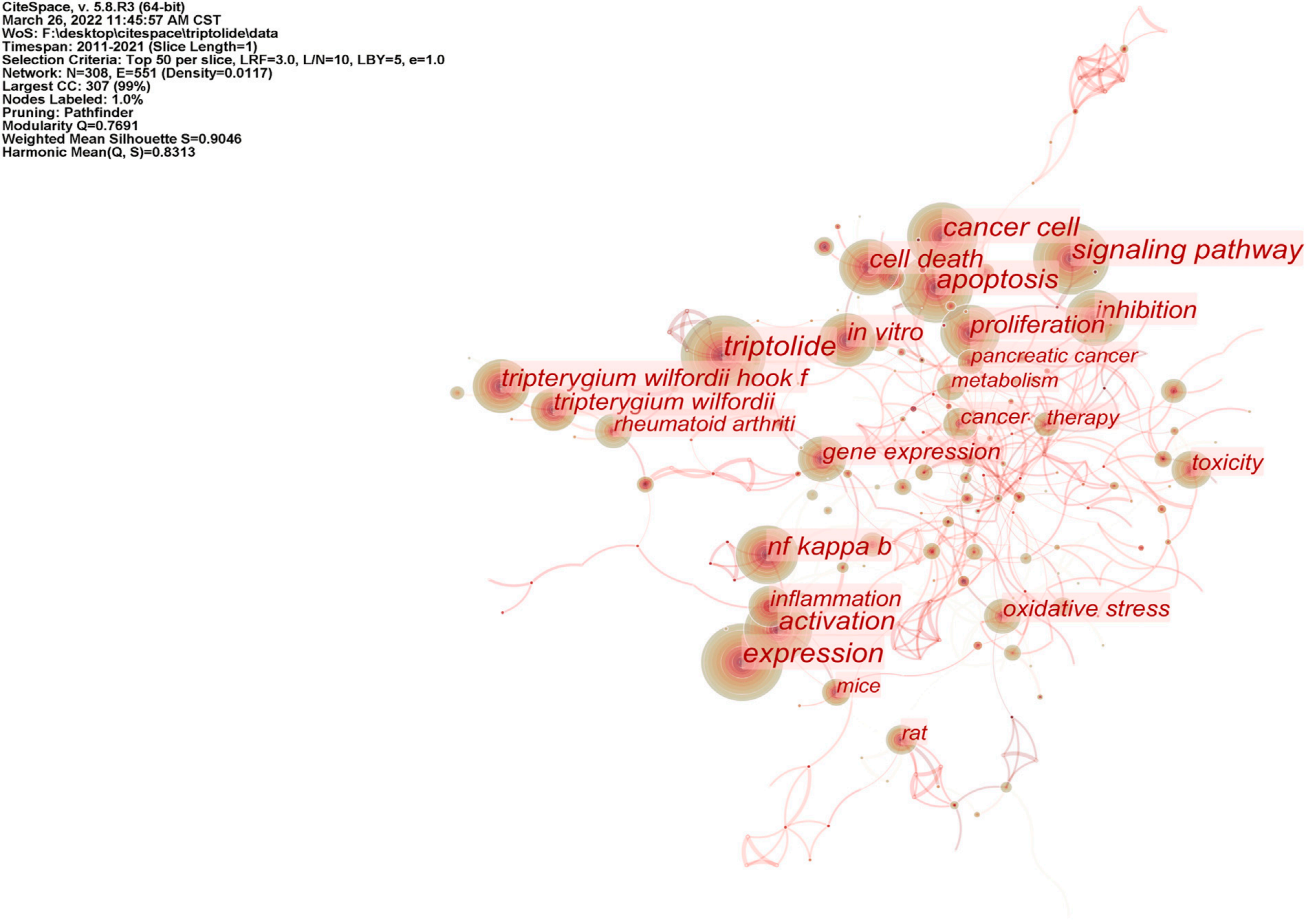
Co-occurrence network of triptolide literature keywords from the WOS.

Based on the visualization analysis of 1,106 English articles, 308 keywords were obtained by combining similar words, and 26 of them had a keyword frequency ≥50. After the visual analysis of the literature keywords, 308 nodes and 1,097 links were obtained. Keyword co-occurrence networks are shown in Figure 5. According to the key words, studies on triptolide in English focus on apoptosis, pharmacological effects, signaling pathways, toxicity, etc. Pharmacological effects involve anti-inflammatory and anticancer activities, mechanisms include oxidative stress and apoptosis, and signaling pathways focus on the NF-κB pathway. Centrality can evaluate the importance of nodes in the network. Nodes with a centrality of more than 0.1 are called critical nodes. Among the keywords of triptolide in the English literature, the highest centricity was TNF-α (0.33), NF-κB (0.25), oxidative stress (0.25), pancreatic cancer (0.21), and induced apoptosis (0.21). It indicates that pharmacological action is the most important research field of triptolide.

**FIGURE 6:**
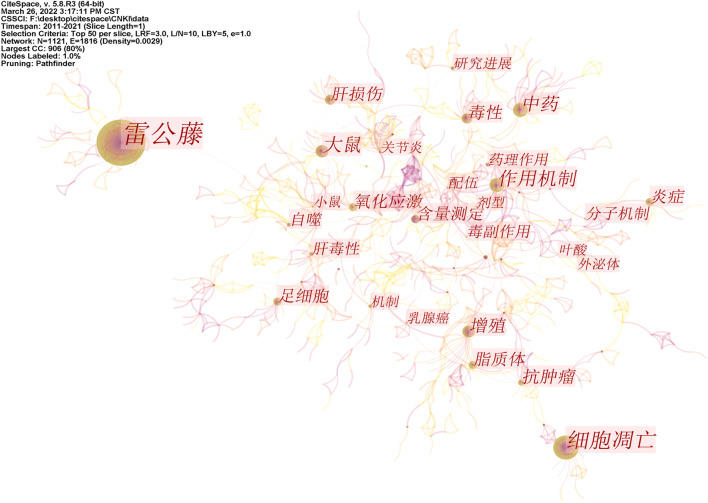
Co-occurrence network of triptolide literature keywords from the CNKI.

Based on the visualization analysis of 1,160 Chinese articles, 1,121 keywords were obtained by combining similar words, and 21 of them had a keyword frequency ≥15. After the visual analysis of the literature keywords, 1,121 nodes and 1816 links were obtained. Keyword co-occurrence networks are shown in Figure 6. According to the keyword information, it can be found that Chinese studies on triptolide focus on apoptosis, anti-tumor effect, toxicity study, inflammatory response, and other aspects. Centrality can evaluate the importance of nodes in the network. Nodes with a centrality of more than 0.1 are called critical nodes. Among the keywords of triptolide in the Chinese literature, the highest centrality was compatibility attenuated (0.24), triptolide (0.23), high-performance liquid phase (0.18), metabolism (0.14), and hepatotoxicity (0.14). The toxicity and compatibility attenuated study of triptolide was emphasized.

#### 2.3.4 Cluster Analysis

**FIGURE 7:**
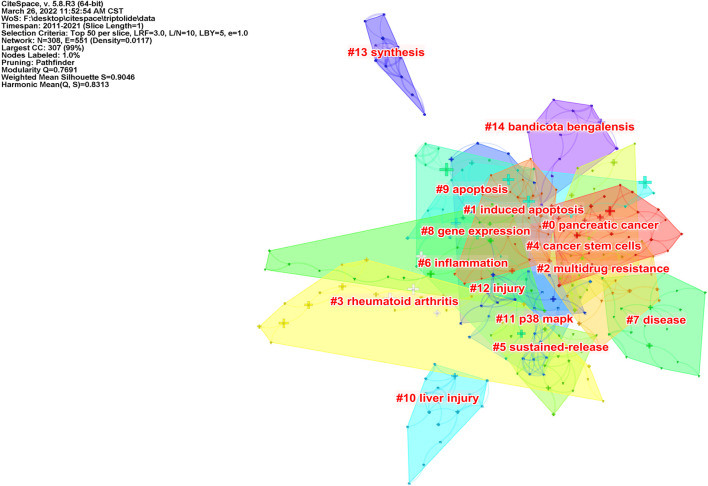
Clustering network of triptolide literature keywords from the WOS.

According to the co-occurrence of keywords, the LLR algorithm was used to extract keywords, and a cluster analysis was carried out. After visualization of the English literature, there are 308 nodes and 1,097 links, forming 15 cluster labels, as shown in Figure 7. The clustering module value *Q* = 0.7691 (Q > 0.3) means that the clustering structure is significant, and the average clustering contour value *S* = 0.9046 means that the clustering result is reliable. Cluster #0, cluster #1, cluster #4, and cluster #9 indicated the significant effect of triptolide in the field of cancer treatment, and clusters #10 and #12 focused on the toxicity of triptolide. Clusters #3 and #6 focused on the study of triptolide in the field of immunity and inflammation.

**FIGURE 8:**
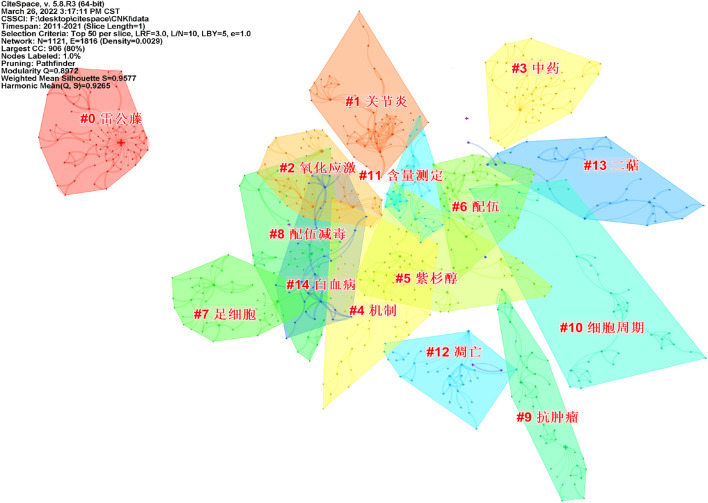
Clustering network of keywords from the CNKI.

After visualization of the Chinese literature, there are 1,121 nodes and 1,816 links, forming 15 cluster labels, as shown in Figure 8. The clustering module value *Q* = 0.8972 (Q > 0.3) means that the clustering structure is significant, and the average clustering contour value *S* = 0.9577 means that the clustering result is reliable. Clusters #9, #10, and #12 indicated the significant effect of triptolide in the field of cancer treatment, and clusters #6 and #8 focused on the toxicity of triptolide. Clusters #1 and #2 focused on the study of triptolide in the field of immunity and inflammation.

**FIGURE 9:**
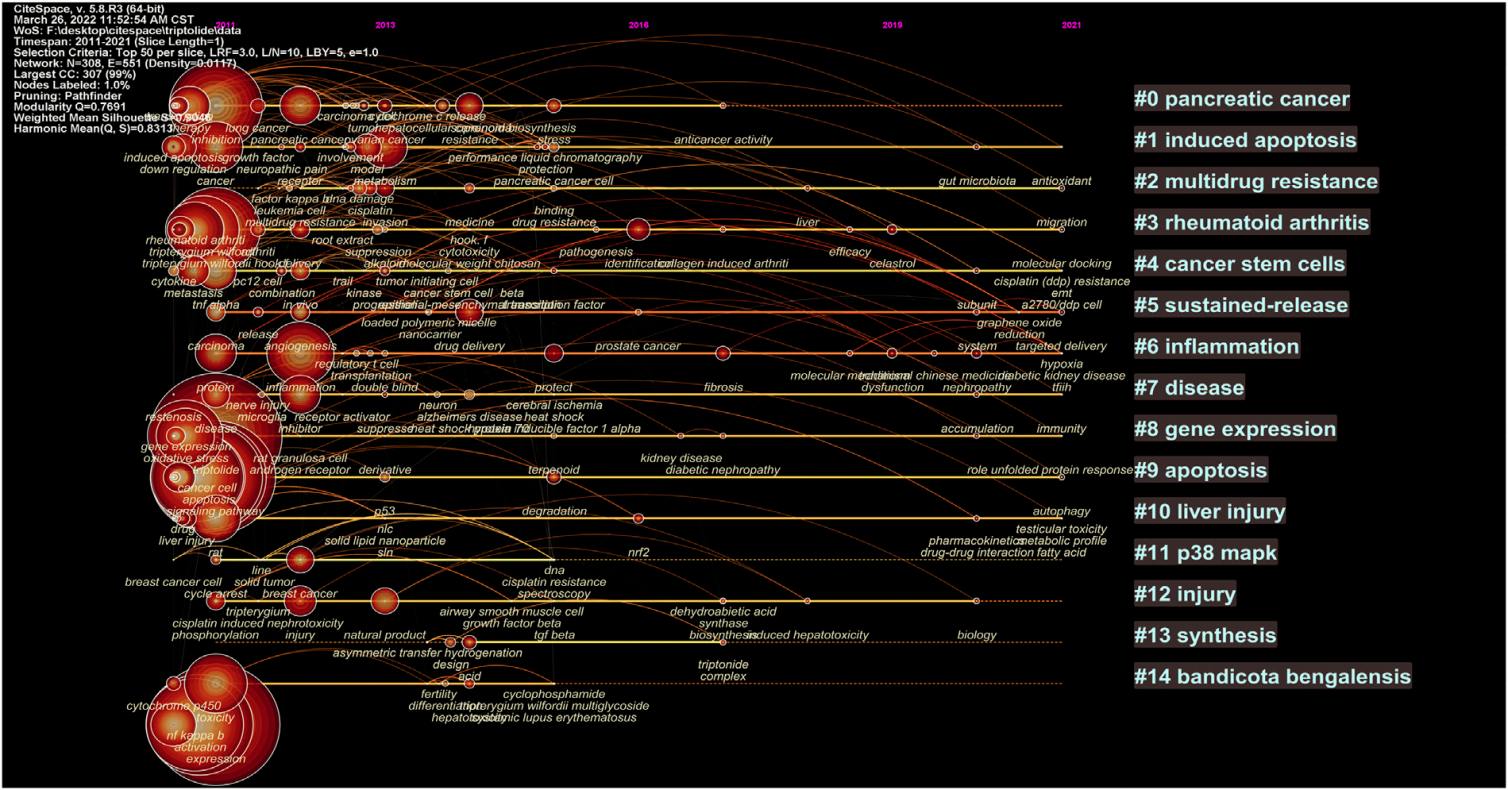
Timeline of the cluster network of triptolide literature keywords from the WOS.

**FIGURE 10:**
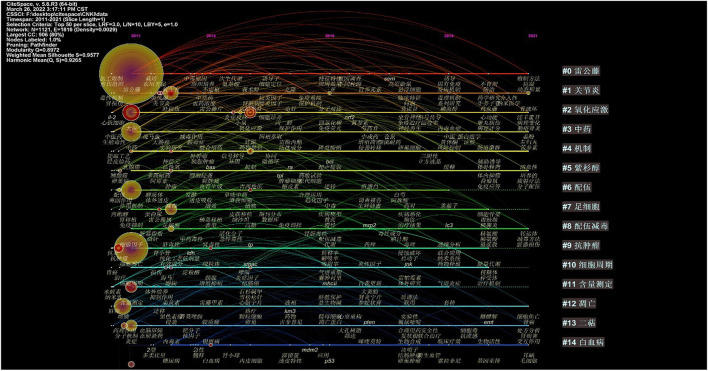
Timeline of the cluster network of keywords from the CNKI.

Keywords contained in the cluster are expanded along the time axis in the TimeLine chart to show the development of the cluster over time, as shown in Figure 9, 10.

#### 2.3.5 Co-Citation Analysis

**FIGURE 11:**
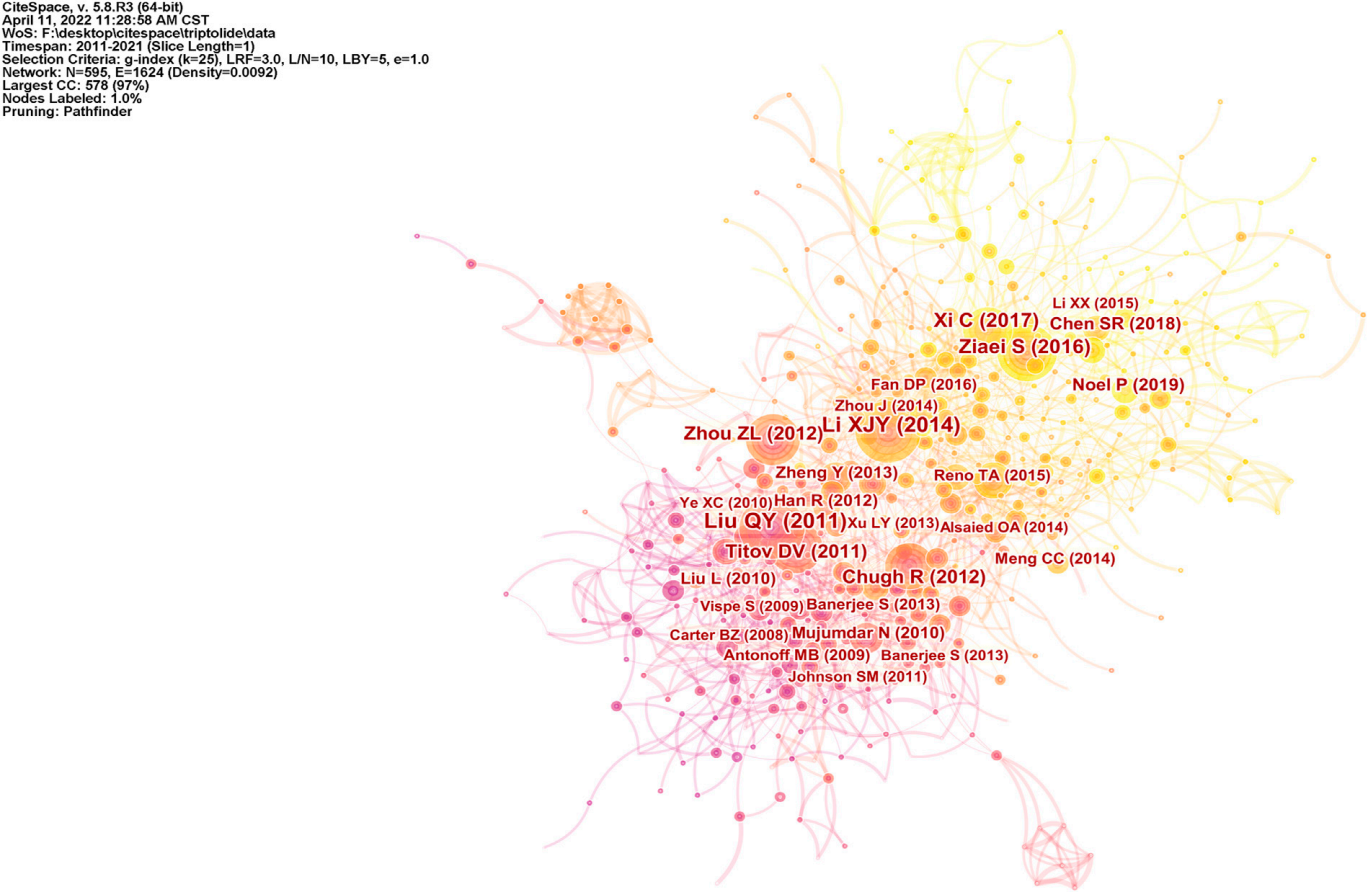
Document co-citation analysis from the WOS.

A total of 1,106 studies from the WOS were analyzed using CiteSpace software. Studies published between 2011 and 2021 were selected for analysis over a one-year time slice, and the most cited or recurring items from each slice were selected. Figure 11 shows a document co-citation network diagram with 595 nodes, and 1,624 links.

The top-ranked item by citation counts is Li et al. (2014) with a citation count of 103, followed by Liu. (2011) with a citation count of 94, Xi et al. (2017) with a citation count of 66, and Zhou et al. (2012) with a citation count of 59. Table 2 shows the top 14 references with the strongest citation bursts. The first three references highlight the emerging trend of triptolide research in 2011, while the middle nine references highlight the emerging trend for 2012–2019. The last two references were those which received great attention in 2019 and continued to 2021, and which are the focus of current triptolide research. Brinker et al. summarized the medicinal chemistry and pharmacology of *Tripterygium wilfordii.* The significant effects of triptolide, its main active ingredient, on anti-inflammatory and autoimmune diseases were emphasized, and the biological activities of terpenoids in addition to triptolide were also mentioned (Brinker et al., 2007). Subsequently, in the report of Li et al. (2014), the antitumor effect of triptolide was mainly introduced as its pharmacological activities, and Meng et al. (2014) even reviewed its role in the field of tumor therapy separately. Phillips et al. (2007) and Mujumdar et al. (2010) reported that triptolide causes pancreatic cancer cell death *in vitro* and *in vivo* by the induction of apoptosis. Phillips et al. (2007) underlined that its mechanism of action is mediated *via* the inhibition of Heat Shock Protein 70 while Mujumdar et al. (2010)’s study focused on triptolide-inducing apoptosis and autophagy. The results of Antonoff et al. (2009) on neuroblastoma confirmed this phenomenon. Chugh et al. (2012) tested Minnelide, a water-soluble form of triptolide, both *in vitro* and in multiple preclinical models of pancreatic cancer. This preclinical evaluation adds theoretical support and confidence to the clinical application of triptolide. Carter et al. (2008)’s results revealed that triptolide could synergistically enhance TRAIL-induced cell death in various leukemia cells by reducing XIAP, a resistance factor to this process, and activating the P53 pathway. As mentioned in the study by Liu (2011), the combination of triptolide and TRAIL therapy may be a key mechanism for overcoming apoptotic resistance. Titov et al. (2011) demonstrated the effect of triptolide on transcriptional activity in A549 cells, and further identified the XPB subunit of transcription factor TFIIH as a key target of triptolide. Particularly noteworthy is that the common inhibition of transcription mediated by RNA polymerase II may be the basis of proliferation inhibition of almost all cancer cells. It should not be ignored that Xi et al. (2017)’s study summarized the toxicity-related mechanism of triptolide with oxidative stress as typical. These results provide some ideas for the study on the structure modification, dosage form modification, and drug combination of triptolide to reduce toxicity and increase efficiency.

**TABLE 2:** Top 14 references with the strongest citation bursts.

References	Year	Strength	Beginning	End	2011–2021
Brinker et al. (2007)	2007	9.42	2011	2012	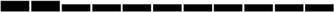
Phillips et al. (2007)	2007	9.42	2011	2012	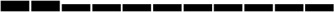
Carter et al. (2008)	2008	8.91	2011	2013	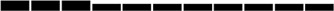
Liu (2011)	2011	21.36	2012	2016	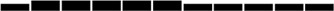
Titov et al. (2011)	2011	12.41	2012	2016	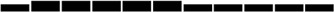
Antonoff et al. (2009)	2009	9.41	2012	2014	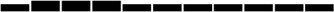
Mujumdar et al. (2010)	2010	9.38	2012	2015	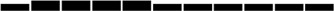
Zhou et al. (2012)	2012	14.52	2014	2017	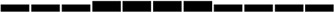
Chugh et al. (2012)	2012	14.23	2014	2017	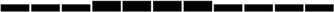
Han et al. (2012)	2012	8.84	2015	2017	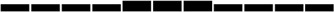
Li et al. (2014)	2014	19.62	2016	2019	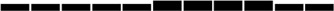
Meng et al. (2014)	2014	8.9	2017	2019	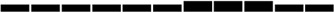
Xi et al. (2017)	2017	20.37	2018	2021	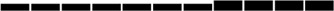
Chen et al. (2018)	2018	14.59	2019	2021	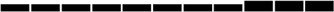

According to the clustering and timeline plot, the anti-tumor studies of triptolide are increasing gradually, especially in breast cancer. Along the timeline, the mechanisms of other cancers, such as colorectal cancer and NSCLC, have been gradually increased. Given the universality and severity of non–small cell lung cancer, the latest research progress on triptolide on NSCLC was summarized in this study.

## 3 Mechanism of Triptolide on Non–Small Cell Lung Cancer

### 3.1 Search Strategies

For this review, research articles on the treatment of NSCLC with triptolide were collected from PubMed and the Web of Science database. Studies were searched with the keywords “triptolide” and “lung cancer.” The literature was included using the following criteria: 1) only articles written in English were included; and 2) these studies should be research papers used to evaluate the efficacy of triptolide in the treatment of NSCLC. Titles and abstracts of all publications retrieved were reviewed to select potentially eligible studies. From a total of 167 results, 53 duplicate studies were removed, and a total of 41 articles were included after Chinese literature, reviews, and other irrelevant literature were removed.

### 3.2 Triptolide Inhibits Non–Small Cell Lung Cancer by Targeting the NF-κB Signaling Pathway

Nuclear factor kappaB (NF-κB) refers to a family of transcription factors including NF-κB1 (p50/p105), NF-κB2 (p52/p100), RelA (p65), c-Rel, and RelB (Dolcet et al., 2005; Zinatizadeh et al., 2021), involved in regulating the expression of genes in many processes such as immunity, inflammation, cell proliferation, migration, and apoptosis, and play a key role in the occurrence and development of cancer. Curcumin (Ghorbanzadeh et al., 2022), ginsenosides (Sun et al., 2021), isoliquiritin (Lv et al., 2021), and other natural active ingredients derived from traditional Chinese medicine have been proven to inhibit the NF-κB pathway. Lee et al. (2002) found that triptolide can inhibit the reverse transcription activation of p65 in A549 cells and NCI-H1299 cells, which is closely related to the activation of NF-κB, and finally allows cells sensitive to TRAIL-induced apoptosis. However, the mechanism by which triptolide inhibits p65 reverse transcription activation is still unclear. The results of the study by Jiang et al. (2016) showed that triptolide inhibited the nuclear translocation of NF-κB in the paclitaxel-resistant human lung adenocarcinoma cell line A549/Taxol, and downregulated the expression of P65 and FLIP, XIAP, Bcl-2, Bcl-XL, and COX-2 genes related to multidrug resistance. This suggests the potential of triptolide combined with anti-cancer drugs such as paclitaxel to reverse conversion therapy drugs to produce drug resistance. Zheng et al. (2017) further found that the status of the p53 gene affects the inhibition of TNF-α-induced NF-κB nuclear translocation by triptolide, that is, triptolide is only significantly inhibited in H460 cells containing the p53 gene NF-κB nuclear translocation, and the effect is greatly reduced in NCI-H1299 and PC-3 cells that do not contain the p53 gene. This is also corroborated in the study by Kumar et al. (2016).

### 3.3 Triptolide Inhibits Non–Small Cell Lung Cancer by Targeting microRNAs

MicroRNAs (miRNAs) refer to small non-coding RNAs that regulate gene expression after transcription (Shah and Shah, 2020). They are used as cancer biomarkers and potential therapeutic targets and are involved in tumor cell proliferation, invasion, growth, and apoptosis process (Shi et al., 2021). A number of studies have shown that triptolide acts on microRNAs to interfere with the proliferation, migration, and apoptosis of colon cancer, breast cancer, non–small cell lung cancer, and other tumor cells. Reno et al. (2015) studied the changes in the expression profile of miRNAs in H460 cells after triptolide induction and the results showed that 126 and 101 miRNAs were significantly upregulated and downregulated. By Ingenuity Pathway Analysis, gene expressions and cellular movements are the altered cellular and molecular functions of the genes that are regulated by the top differentially regulated miRNAs. Next, triptolide was shown to inhibit the migration, invasion, and metastasis of cancer cells *in vivo* and *in vitro*. Li et al. (2016) found that triptolide treatment reduced the expression of miR-21 in PC-9 cells and enhanced the expression levels of phosphatase and tension homolog (PTEN). In addition, upregulating the expression level of miR-21 inhibited the effect of triptolide on the viability of PC-9 cells and the expression level of the PTEN protein. The study of Philips et al. (2020) showed that triptolide upregulated the expression of miR204-5p, which led to the downregulation of Cav-1 and Sirt-1 mRNA expression in human A549/NCI-H460 cells, and activated the classic mitochondrial apoptosis pathway mediated by Akt/Bcl-2, and led to the apoptosis of A549 and NCI-H460 cells.

### 3.4 Triptolide Inhibits Non–Small Cell Lung Cancer by Targeting the MAPK Signaling Pathway

Mitogen-activated protein kinases (MAPKs) are key regulatory factors involved in cell growth, movement, survival, and apoptosis in physiological and pathological processes (Dhillon et al., 2007; Liu et al., 2018). There are three major MAPK groups in mammals: extracellular signal-regulated protein kinases (ERK1/2), p38MAP kinases (subtypes α, β, γ, and δ), and C-Jun amino-terminal kinases (JNK1/2/3) (Low and Zhang, 2016). The MAPK signal cascade consists of at least three hierarchical kinase components: MAPKK kinase (MAPKKK), MAPK kinase (MAPKK), and MAPK. MAPKKKs phosphorylate and activate MAPKKs, which in turn phosphorylate and activate MAPKs (Kim and Choi, 2015). The occurrence and development of a variety of human cancers involve the disorder or damage of the MAPK signaling pathway, which is mainly caused by genetic and epigenetic changes (Asl et al., 2021). Frese et al. (2003) found that tumor cells (A549 and NCI-H358 cell lines) that were resistant to apoptosis induced by the Apo2L/TRAIL ligand could be sensitized by triptolide. In addition, the activation of ERK2 was observed. At the same time, compared with p38 inhibitors, only ERK inhibitors can block this sensitization, suggesting that ERK2 is essential for triptolide-mediated sensitization. More than 90% of cancer patients treated with traditional chemotherapy agents or newly targeted agents die from multidrug resistance (MDR) (Bukowski et al., 2020). Considering that MDR is a major barrier to effective chemotherapy for cancer, Xie et al. (2016) studied the anti-proliferation effect of triptolide on the paclitaxel-resistant cell line A549/Taxol, and used different MAPK inhibitors to explore the effect of triptolide on the MAPK signaling pathway. Consistent with previous studies, the P38 inhibitor SB202190 had little effect on the anti-proliferation activity of triptolide, while JNK inhibitor SP600125 and ERK inhibitor U0126 had significant antagonistic and synergistic effects on triptolide, respectively. Western blots showed that triptolide increased the expression of P-P38, P-ERK, and P-GSK-3β, and downregulated the expression of P-JNK and P-Akt, suggesting that triptolide plays a pro-apoptotic and anti-proliferative role by regulating JNK and ERK signaling pathways. In addition, Tai et al. (2010) also found that triptolide could reverse the reduction of rosiglitazone-mediated cell invasion and migration, and downregulate the expression of MKP-1. These results suggest that triptolide may be involved in the regulation of multiple pathways of the MAPK signaling cascade, thus interfering with several key processes such as proliferation, apoptosis, migration, and invasion in NSCLC.

### 3.5 Triptolide Inhibits Non–Small Cell Lung Cancer by Targeting the Wnt/β-Catenin Signaling Pathway

The Wnt/β-catenin signaling pathway is an evolutionarily conserved pathway that plays a key role in embryonic development and is involved in the regulation of adult stem cell homeostasis and tissue regeneration. The activation of the Wnt/β-catenin pathway is closely associated with many cancers and diseases including non–small cell lung cancer. In the absence of Wnt, β-catenin is bound and regulated by a degradation complex composed of the scaffold protein (AXIN), adenomatous polyposis coli (APC), casein kinase 1α(CK1α), and glycogen synthase kinase 3β (GSK 3β) (Chatterjee et al., 2021). This complex further leads to phosphorylation, ubiquitination, and proteasome decomposition of β-catenin, maintaining a low expression of intracellular dissociative β-catenin. After activation by Wnt, β-catenin was rapidly enriched in the cytoplasm and transferred to the nucleus. Nuclear β-catenin, as a coactivator of T-cell factor/lymphoid enhancer factor (TCF/LEF) (Yu et al., 2021), activates the transcription of downstream Wnt genes.

Wnt inhibitory factor-1 (WIF1) is a typical Wnt antagonist (Guo et al., 2017) and can bind to the Wnt protein to inhibit the Wnt/β-catenin signaling pathway. A number of studies have shown that WIF1 is downregulated by hypermethylation of promoter regions in lung cancer tissue, resulting in the abnormal activation of the Wnt signaling pathway and participating in the development of lung cancer. Reno et al. (2016) found that the triptolide derivative MRx102 can significantly reduce the proliferation of cell lines H460 and A549, stimulate cell apoptosis, and effectively inhibit their chemotaxis migration and invasion. WIF1 expression was significantly increased in MRx102 -treated mouse tumor tissues. Mao et al. (2018) evaluated the effect of triptolide on WIF1 in non–small cell lung cancer by methylation-specific PCR detection, and the results showed that triptolide could significantly induce WIF1 demethylation in lung cancer cell lines A549 and H460. Inspired by this part of the study, Nardi et al. (2018) further evaluated the effects of triptolide on five Wnt inhibitors WIF1, FRZB, SFRP1, ENY2, and DKK1 in NSCLC. The results showed that triptolide treatment induced significant upregulation of five Wnt inhibitors in A549 and H460 cells. Further experiments showed that triptolide induced an overall reduction in the methylation of key residues of histone H3, including H3K4, H3K9, H3K27, H3K36, and H3K79. These results are of great importance for the study of the association between triptolide and the Wnt/β-catenin pathway, and provide a new idea for the development of triptolide as a new epigenetic modifier targeting the Wnt/β-catenin pathway.

Metastasis is the leading cause of death in patients with NSCLC, epithelial-mesenchymal transition (EMT) is considered to be a key mechanism in cancer progression and metastasis (Singh et al., 2018). Studies have shown that triptolide can regulate EMT in cancer progression by inhibiting the Wnt/β-catenin pathway, thus inhibiting the migration and invasion of tumor tissues (Liu et al., 2015; Basu et al., 2018). Deng et al. (2021) found that after triptolide treatment, the expression of the epithelial marker E-cadherin in NCI-H1299 cells was significantly increased, while the expressions of mesenchymal markers ZEB1, vimentin, and slug were decreased, and the expression of β-catenin was also inhibited. The researchers overexpressed and knocked out genes in NCI-H1299 and NCI-H460 cells, respectively, and β-catenin confirmed that inhibition of EMT is mediated by β-catenin. Tian et al. (2021) explored the in-depth mechanism of triptolide affecting β -catenin and the results showed that triptolide-induced β-catenin degradation is regulated by glycogen synthase kinase GSK-3 and p70S6K dephosphorylation in A549/TaxR cells. Triptolide activates GSK-3 by blocking the activity of p70S6K, promotes the degradation of β-catenin, and inhibits the Wnt/β-catenin pathway.

### 3.6 Triptolide Inhibits Non–Small Cell Lung Cancer by Targeting Tumor Suppressor p53

Tumor suppressor p53 is encoded by TP53 (Blagih et al., 2020) and is involved in regulating cell cycle progression, apoptosis, cell senescence, and other key processes that control normal cell growth and death (Chappell et al., 2016; McCubrey et al., 2017). Mutations, gene deletions, and increased expression of negative regulators (such as MDM2 or MDM4) all lead to the damage or loss of p53 function, which is common in human malignant tumors. For example, TP53 is mutated in 50% of invasive tumors (Duffy et al., 2017). Therefore, it is of great significance to develop targeted cancer therapies that activate and stabilize p53 signaling pathways (Huang, 2021). Kumar et al. (2016) evaluated the cytotoxicity of triptolide in lung cancer cell lines with different p53 states. The results showed that compared with A549 and NCI-H460 cell lines with wild-type p53, the cell viability of NCI-H1299 and NCI-H2009 cell lines with p53 deletion was significantly reduced at similar doses. Wang et al. (2020) found that triptolide treatment increased the binding of the ribosomal protein L23 (RPL23) to MDM2 and weakened the binding of MDM2 to p53, directly leading to the reduction of MDM2-mediated degradation of p53. Apoptosis and cell cycle arrest can be induced by activating p53, upregulating apoptosis regulators caspase9 and caspase3, and inhibiting Bcl-2. In addition, upregulation of p53 was also observed in other studies on triptolide (Zheng et al., 2017; Mao et al., 2018).

### 3.7 Triptolide Inhibits Non–Small Cell Lung Cancer by Targeting Other Signaling Pathways

Akt is a serine/threonine kinase and a carcinogenic protein that regulates cell survival, proliferation, growth, apoptosis, and glycogen metabolism. It is involved in the PI3K/Akt signaling pathway (Song et al., 2019). Akt is the central node of a large number of signaling pathways by signaling upstream regulatory proteins such as PTEN, PI3K, and receptor tyrosine kinases to many downstream effectors such as GSK-3β, FOXO, and MDM2 (Revathidevi and Munirajan, 2019). Xie’s results showed that the expression of P-Akt and P-GSK-3β in A549/Taxol cell lines was significantly upregulated after triptolide treatment, which was also observed by Yang et al. (2011), Meng et al. (2015), and Tong et al. (2019). Considering the specificity of Akt as the central node, this may partially explain the involvement of triptolide in NF-κB, p53, and Wnt/β-catenin signaling pathways in the regulation of NSCLC progression.

STAT3 refers to the signal transduction and transcriptional activator 3. Abnormal activation of STAT3 triggers tumor progression through oncogene expression, leading to a malignant tumor (Lee et al., 2019). STAT3 activation is often found in solid cancers, including NSCLC. In NSCLC patient samples, a high p-STAT3 level is associated with advanced disease, smoking, and EGFR status, and can be used as a marker to predict patient survival (Mohrherr et al., 2020). Huang et al. (2019) found that triptolide treatment can reduce STAT3 phosphorylation in PC-9 and A549 cell lines, inhibit STAT3 entry into the nucleus, and reduce the expression of C-Myc, Bcl-2, and matrix metallopeptidase 9 (MMP-9), and these STAT3 target genes are involved in cell survival, apoptosis, and migration. In addition, interleukin-6 (IL-6)-induced activation of STAT3 target genes such as McL-1 and Bcl-2 can be attenuated by triptolide.

The Keap1-Nrf2 signaling pathway plays a key role in the oxidative stress response of lung cancer. Keap1 refers to the Kelch-like ECH-related protein, and Nrf2 is the E2-related factor 2 encoded by NFE2L2 (Taguchi and Yamamoto, 2017). The continuous activation of Nrf2 in lung cancer cells is generally due to the mutation of the Keap1/NFE2L2 gene, thus resulting in drug resistance and significant invasiveness (Zhang et al., 2019). A study by Zhu et al. (2018) indicated that the non-cytotoxic concentration of triptolide reduced mRNA levels of NFE2L2 and its downstream genes GCLC and AKR1C1 in A549 cells, and inhibited Nrf2 protein levels. Triptolide also inhibited the Nrf2-ARE pathway *in vivo*.

A common feature in the different signaling pathways mentioned previously is that a large number of transcription factors and cell cycle regulators are downregulated. Triptolide has been reported to have global transcriptional inhibitory activity (Leuenroth and Crews, 2008; Vispe et al., 2009). Wang et al. (2011) confirmed that triptolide induced phosphorylation and subsequent proteasome-dependent degradation of Rpb1, the largest subunit of RNA polymerase Ⅱ. This may be the key for triptolide to exert its potent antitumor activity against NSCLC and even a wide range of human cancers.

## 4 Potential of Triptolide in Drug Combination

Clinical studies have shown that triptolide exposure can cause significant organ damage in humans, including hepatotoxicity, nephrotoxicity, cardiotoxicity, and reproductive toxicity (Xi et al., 2017). Accordingly, a large number of studies on the toxicity of triptolide have been carried out *in vitro* and *in vivo* to clarify its toxicological mechanism and dose-dependent characteristics and widen its application scope. Presently, studies on the application of triptolide are mainly divided into two categories. One kind of study is to develop targeted delivery systems for triptolide or modify its structure, including the use of nano-carriers and the design of antibody conjugation drugs (Zhang et al., 2020), to reduce toxicity and improve its clinical application potential. The other, because of the broad-spectrum anticancer activity of triptolide against multiple tumors with multiple targets and multiple pathways, it is suggested that triptolide combined with other drugs at low doses may play a significant anticancer role while reducing its toxicity and side effects. The following are studies on the inhibition of non–small cell lung cancer by triptolide combination.

EGFR mutations are typical carcinogenic drivers in NSCLC and have been observed in about 50% of Asian patients (Harrison et al., 2020). Epidermal growth factor receptor-tyrosine kinase inhibitors (EGFR-Tkis) represented by gefitinib have shown good efficacy as adjuvant therapy for early NSCLC patients with EGFR-sensitized mutations (Ye et al., 2021). Tong et al. (2019) found by MTT analysis and combination index (CI) analysis that gefitinib, erlotinib, and icotinib had synergistic effects with triptolide at different concentrations (5 and 15 μm) on H1975 cell lines. However, there was no synergistic effect on the H1299 cell line. It is worth noting that increasing the concentration of triptolide has little effect on the synergistic effect, which is conducive to the anticancer effect of triptolide combined with other drugs at low toxic concentrations in view of the dose-dependent characteristics of triptolide toxicity. Li et al. (2020) also found that the abnormal expression of E-cadherin in gefitinib-sensitive cells induced gefitinib resistance. Triptolide effectively increased the sensitivity of drug-resistant A549 cells to gefitinib by upregulating the expression of E-cadherin and downregulating the expression levels of MMP-9 and vimentin, which could overcome this problem.

Cisplatin-based combination chemotherapy is the standard first-line therapy for patients with unresectable advanced NSCLC (Bluthgen and Besse, 2015), and platinum compounds are considered to be the most effective anticancer drugs in the clinical treatment of NSCLC at present. However, resistance to cisplatin can be induced by multiple factors (Rose et al., 2014; Kryczka et al., 2021), and resistance to cisplatin in lung cancer is a key factor affecting the therapeutic effect. Zhu et al. (2018) found that triptolide at non-cytotoxic concentrations increased the sensitivity of A549 cells to cytotoxic chemotherapeutic agents (cisplatin, etoposide, and erythromycin) and triptolide increased the chemotherapy sensitivity of xenotransplanted tumors *in vitro*. Reno et al. (2016) also found a synergistic effect between triptolide derivative MRx102 and carboplatin in an H460 xenograft tumor mouse model.

## 5 Discussion

For a long time, the high incidence and mortality of lung cancer has been the main reason causing the death of cancer patients. Drug resistance and metastasis have brought great challenges to the effective treatment of NSCLC, and there is an important need for effective and safe new drugs and treatment strategies. In this study, CiteSpace was used to conduct a visual analysis of triptolide research based on 1,106 English literature and 1,160 Chinese literature data on triptolide research retrieved from the WOS core collection and CNKI from 2011 to 2021. Through the exploration of keyword clustering, the first is the significant therapeutic effect of triptolide in the fields of immune inflammation and cancer treatment. It is worth noting that the toxin study of triptolide should not be ignored, which is also the significant feature that distinguishes its clinical application from other traditional Chinese herbal active ingredients. Through a visual knowledge spectrum, this study helps to enrich the existing knowledge system of triptolide research worldwide. It provides valuable guidance for researchers and related personnel.

As a traditional Chinese medicine with a long history of application, *Tripterygium wilfordii* was initially used to treat rheumatoid arthritis and other immune inflammation. Accordingly, the effect of triptolide on immune inflammation was not disappointing, but its clinical application was limited by its serious organ and reproductive toxicity. Interestingly, the remarkable anticancer activity found in triptolide in recent years has given it a new lease of life. This article reviews the current research progress on the anti-NSCLC effects of triptolide. The anticancer activity of triptolide involves multiple signaling pathways, including the NF-κB pathway, microRNAs, MAPKs, Wnt/β-catenin pathway, etc. These studies have demonstrated the clinical therapeutic potential of triptolide, with special emphasis on apoptosis, EMT, and gene expression regulation. In particular, the signaling pathway represented by NF-κB is highly correlated with immunity and inflammation. In addition, studies have shown that Wnt/β-catenin can affect the immune invasion of tumor tissues, which is expected to become a new tumor immunotherapy target (Spranger et al., 2015). There have been studies on the immunomodulatory effects of triptolide on mesenchymal stromal cells (He et al., 2021). However, no extensive studies have been conducted on the effects of triptolide on the immunomodulatory microenvironment of NSCLC. It is foreseeable that triptolide may provide a new idea for immunotherapy of NSCLC based on its superior immune activity.

In addition, because of the toxicity and poor water solubility of triptolide, some studies on drug combination were summarized. Beyond that, structural modifications and nano-drug delivery systems are also common strategies. Minnelide is synthesized from triptolide, which has been used in clinical trials (Chugh et al., 2012). Studies have been conducted to improve the pharmacokinetics and toxicity of NSCLC by designing triptolide-associated antibody conjugants that target NSCLC (Zhang et al., 2020). However, to truly apply triptolide in the treatment of NSCLC, further research on triptolide and its derivatives and new dosage forms are needed to truly apply triptolide in the treatment of NSCLC. At the same time, the metabolic characteristics of triptolide *in vivo* should also be paid attention to, and even the concentration monitoring of triptolide should be done to guide the individualization and safety.
